# Effects of walking on bilateral differences in spatial attention control: a cross-over design

**DOI:** 10.1186/s40945-015-0012-y

**Published:** 2015-10-23

**Authors:** Soichiro Koyama, Shigeo Tanabe, Yuichi Hirakawa, Hiroaki Sakurai, Yoshikiyo Kanada

**Affiliations:** 1 Department of Rehabilitation, Kawamura Hospital, 501-3144 Gifu, Japan; 2 Faculty of Rehabilitation, School of Health Sciences, Fujita Health University, 470-1192 Toyoake, Japan

**Keywords:** Stimulus–response compatibility task, Inhibitory function, Visual special attention, Walking

## Abstract

**Background:**

Walking requires a high attentional cost for balance control and interferes with the control of attention. However, it is unclear whether the performance of visual spatial attention control, which is one of the functions of attention control, is also decreased during walking. In addition, although previous studies have shown right-hemispheric dominance and lower ability of left side visual spatial attention control during sitting, it remains unknown whether walking accentuates bilateral differences in visual spatial attention control. We tested the hypothesis that walking interferes with visual spatial attention control on both sides and accentuates its bilateral differences.

**Methods:**

Twenty healthy right-handed subjects (24.3 ± 2.0 years) participated in this study. Subjects performed a random stimulus–response compatibility (SRC) task during both sitting and walking situations. To evaluate the effects of walking, reaction time was measured on both sides for the two situations. In comparison to the both situations (sitting and walking), the amount of change of the SRC effect on both sides was used. In the comparing the bilateral difference (left and right), the difference of the SRC effect was evaluated in each situation. The paired t-test was applied to both comparisons for statistical analysis.

**Results:**

The SRC effect on both sides during walking was significantly larger than during sitting (*P* < 0.05). In addition, walking significantly accentuated the bilateral differences in visual spatial attention control (*P* < 0.05).

**Conclusions:**

These results suggest that walking affects the performance of visual spatial attention control on both sides and accentuates its bilateral differences. These results have implications for development of practice methods of gait disorder with higher brain dysfunction.

## Background

Although the control of attention is a critical ability during walking [1], walking requires a higher attentional cost for balance control compared to during sitting and quiet standing [2] and interferes with the control of attention. During walking, the simultaneous execution of an attention task (e.g., digit span task, Stroop task, and selective reaction time task) causes a decline in performance in various populations including healthy young adults, older adults, and patients with neurologic disease [3–8]. However, it is unclear whether the performance of visual spatial attention control, which is one of the functions of attention control, is also decreased during walking.

In addition, spatial attention control has bilateral differences that are a fundamental feature in humans, whether young, elderly or diverse patient populations [9–11]. As a neurophysiological mechanism, the right-hemispheric cortex controls visual spatial attention control in both visual hemifields (i.e., the right as well as the left visual hemifield), whereas the left-hemispheric cortex attends to the right visual hemifield only [12]. Empirical findings from brain-damaged patients and functional brain imaging studies argue for right-hemispheric dominance [13–15]. Although bilateral differences in visual spatial attention control have been demonstrated during sitting [16], the effects of walking on these differences have not been shown.

Our hypothesis is that the performance of visual spatial attention control is decreased during walking because of the attentional cost for balance control. In addition, bilateral differences in visual spatial attention control are accentuated during walking because the effect of the weaker side (left space) on visual spatial attention control is more strongly affected. The present research was designed as a preliminary study to test these hypotheses in healthy young adults. Experimental verification of the hypothesis might be useful for the development of treadmill gait training with visual feedback system.

## Methods

### Subjects and experimental setup

Twenty healthy right-handed subjects (24.3 ± 2.0 years) participated in this study. All subjects provided informed consent to the experimental procedure, which was approved by the human ethics committee of our Hospital. This study was performed in accordance with the Declaration of Helsinki.

The present study employed a within-group, cross-over experimental design to test the effects of walking on spatial attention control. All subjects performed a random stimulus–response compatibility (SRC) task [17] during sitting and walking. The order of the situations was counterbalanced across participants to exclude the order effect. In the sitting situation, the subjects were seated comfortably in a chair in front of a computer screen (15 inch) and were required to maintain this position throughout the study. In the walking situation, the subjects walked comfortably (speed range 2.0–2.6 km/h) on a treadmill. A computer screen and keyboard were located at the level of the thorax of each subject in front of the walker. The index and middle fingers of the right hand were set on the arrow buttons of a keyboard in the setup position. The left arrow button was pushed by the index finger, whereas the right arrow button was pushed by the middle finger. During walking on the treadmill, the subjects were allowed to hold a handrail to prevent falling if they were not able to recover from unusual postural change.

### SRC task procedure

The SRC task that was programmed using the open-source package PsychoPy [18] reflects the overlap of spatial properties between stimuli and responses. The subjects were instructed to ignore the location of an arrow, and respond based solely on the direction of the arrow. When the stimulus location of the arrow and the direction of the arrow were congruent (congruent condition), the subjects had to press the button indicating the same direction as the arrow. Conversely, when the stimulus location of the arrow and the direction of the arrow were incongruent (incongruent condition), the subjects had to press according to the direction of the arrow. Before the start of the task, a tip-stimulated black “ready??” was presented centrally on the screen. After pressing the enter key (as the start button), a fixation stimulus consisting of a black “+” was presented centrally for 1000 ms. The subjects were instructed to focus on the fixation stimulus. Following the fixation stimulus, a test stimulus (“<” or “>”) appeared randomly until a response button was pressed. The subjects were asked to respond by pressing the arrow key as fast and correctly as possible. In the congruent condition, the reaction time (RT) from stimulus presentation to response is fast and the responses have high accuracy. In contrast, the RT becomes sluggish and the responses have low accuracy under the incongruent condition [17]. The SRC effect is defined as the difference of RT between the congruent and incongruent conditions, and indicates subjects’ level of selective cognition (paying complete attention to the direction of the arrow, while ignoring the location of the arrow).

In the present study, after receiving instructions on this task, all subjects underwent ten trials of the task to gain familiarity with the task in first experimental situation (sitting or walking situation). If a subject did not understand this task, the experimenter provided an explanation of the task again. Then, each subject received 240 trials (30 trials with respect to 4 conditions, which consisted of 2 locations (left and right fields) of stimulation and 2 directions of the arrow (left and right) × 2 situations). The order of trials was randomized within one situation. The inter-trial interval was 2 s from pressing an arrow key. After finishing one situation (sitting or walking situation), subjects performed same tasks again with other situation.

### Data analysis

To determine to put the SRC task in the proper perspective, rate of correct answers was used. The rate was defined as the number of times a button was missed or wrongly pressed, divided by the total number of trials. In addition, the pearson’s chi-square test was used to assess the equivalently in the rate of correct answers among situations (sitting and walking).

To compare the SRC effect between sitting and walking situations, the amount of change of the SRC effect on both sides was used. Then, to assess the bilateral difference (left and right) of the SRC effect, the results of SRC effect was divided into 2 categories: 1) the SRC effect in the left field and 2) the SRC effect in the right field and compared between categories. In both comparisons, statistical significance was evaluated by using the paired t-test and set a level at P < 0.05. SPSS software (version 19, SPSS, Chicago, IL, USA) was used for statistical analyses.

## Results

All subjects completed the experiment and did not report any side effects. In all trials, every subject could keep a stable posture without holding the handrail. There was no significant difference in the rate of correct answers between sitting (mean ± standard deviation; 95.8 ± 2.1 %) and walking (95.9 ± 2.3 %) (*P* >0.05). This result provides evidence that the RTs between both situations can be compared by excluding effects caused by the rate of correct answers.

In the sitting situation, RT in the congruent condition (416.55 ± 73.56 ms) was significantly faster than in the incongruent condition (437.9 ± 71.42 ms) (*P* < 0.01). Similarly, in the walking situation, RT in the congruent condition (386.38 ± 41.53 ms) was significantly faster than in the incongruent condition (419.21 ± 44.30 ms) (*P* < 0.01).

**Fig. 1:**
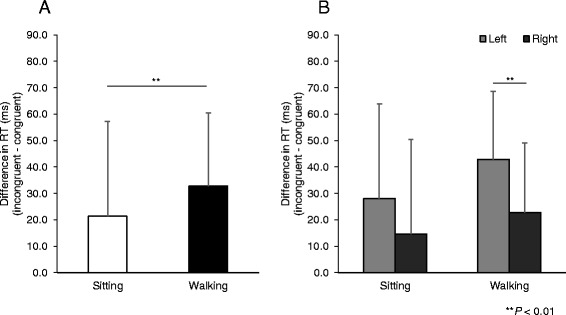
The stimulus–response compatibility (SRC) effect. Data are means and the error bars indicate standard deviation. The left figure shows the differences in reaction time (RT) of each situation. **a** The X-axis is situation (sitting and walking). The Y-axis indicates differences in RT (incongruent − congruent) (ms). The black bar indicates the walking situation, whereas the white bar indicates the sitting situation. The right figure shows the differences in RT of the bilateral differences. **b** The X-axis is situation. The Y-axis indicates differences in RT (incongruent − congruent) (ms). The black bar indicates the right side, whereas the gray bar indicates the left side

The amount of the SRC effect between both situations is shown in Fig. 1a. The SRC effect was 21.36 ± 35.93 ms in the sitting situation and 32.82 ± 27.64 ms in the walking condition. The paired t-test revealed that the SRC effect was significantly larger in the walking situation than in the sitting situation (*P* < 0.01).

Figure 1b shows the bilateral difference of the SRC effect in each situation. The bilateral difference in the sitting situation was not significantly different between the left (28.09 ± 35.74 ms) and right (14.64 ± 35.74 ms) (*P* = 0.14). Conversely, in the walking situation, there was a significant difference between the left (42.89 ± 25.76 ms) and right (22.76 ± 26.29 ms) (*P* < 0.01).

## Discussion

Using the SRC task, the present study tested the hypothesis that walking decreases the performance of visual spatial attention control and accentuates the bilateral difference between the visual fields. RT in a congruent condition is usually reported to be shorter than in an incongruent condition [17]. The present results replicated this previous study in sitting and walking situations. Thus, the effect of walking on visual spatial attention control can be verified from the present results.

The primary results showed that RT during walking was significantly longer than during sitting. Previous studies indicated that the simultaneous execution of walking with various attention tasks decreases walking stability and the performance of attention tasks [3–8]. The present results suggest that visual spatial attention control is also limited during walking.

In addition, the SRC effect in the left field was significantly larger than in the right field during walking, whereas there was no significant difference during sitting. To our knowledge, previous studies have not clarified the effects of the bilateral differences in visual spatial attention control because these studies only employed tasks at the center of the visual spatial field [3–6, 8] or measured RT from somatosensory stimulation [7]. The present results suggest that right-hemispheric dominance on visual spatial attention control becomes prominent during walking.

There are a few limitations to the present study that merit consideration. First, the present study only compared between sitting and walking. Further study should be performed with other movement conditions (e.g., cycling and foot-pad tapping) to investigate whether the present results were caused by walking-specific effects. By comparing between walking and other movements, it might become clear whether the effects are caused by walking-specific or merely motion-specific. Second, the present study could not verify the effects of the intensity of the walking parameters. Previous studies suggested that the performance of a cognitive task is affected by differences in exercise intensity [19–21]. Third, our study used an arrow as the stimulation object in the SRC task. A previous study suggests that the SRC effects elicited by an arrow and location are more related, whereas those elicited by words and location are less related [22]. Thus, a future study should examine whether the effects of walking on the bilateral differences in spatial attention control are dependent on the stimulation object. In addition, the further study should be also performed in other populations such as elderly and patients with neurologic disease for clinical application.

## Conclusions

These results have implications for development of practice methods using treadmill gait training with visual feedback system for gait disorder with higher brain dysfunction such as patients with traumatic brain injury and other patients with decreased attention ability regardless of neglect-like syndrome. Previous studies proposed the treadmill gait training with visual feedback system in patients with neurological diseases [23–25]. In general, visual feedback system generates information centrally on a screen installed in front of the walker. However, no report concerning an effective location of visual feedback has been published although an increase in attentional demand decreases walking stability in patients with walking disability [2]. The present results suggest that the visual feedback required to pay special attention should be displayed in right of the screen.

## Abbreviations

SRC: Stimulus–response compatibility
RT: Reaction time
